# Are We Taking Good Care of Our Patients and Physicians?

**DOI:** 10.5935/abc.20180115

**Published:** 2018-08

**Authors:** Gilson Soares Feitosa

**Affiliations:** 1 Escola Bahiana de Medicina, Salvador, BA – Brazil; 2 Hospital Santa Izabel da Santa Casa da Bahia, Salvador, BA – Brazil

**Keywords:** Medical Assistance, Cardiovascular Diseases, Ambulatory Care, Emergency Medical Services, Inservice Training, Medical Education / manpower

The thorough and well-structured analysis by Marcolino et al.^1^ in the article “Satisfaction of emergency physicians with the care provided to patients with cardiovascular diseases in the Extended Northern Region of Minas Gerais”, published in this *Arquivos Brasileiros de Cardiologia* issue, highlights important problems to be addressed in the Brazilian medical healthcare.

Although circumscribed to a region, that study would most probably reproduce the reality of several other Brazilian regions, some even with a higher human development index. The dissatisfactions brought up in that study can be easily observed in the daily medical practice in almost all Brazilian cities, notably emergency care in general, and emergency cardiovascular care in particular. In the later, the issue is compounded by the frequent need for combining good and prompt care to yield effectiveness.

One of the major problems detected in the care provided to emergency patients is the physicians’ lack of specific training in cardiovascular diseases, which might be the reason of the other finding in the study referred to, the physicians’ dissatisfaction with their work.

It is certainly not reasonable to assume that physicians trained in cardiovascular diseases will be available at all less populated regions of Brazil. Grouping the care provided according to complexity, with an agile referral system between units from lower to higher capacity for care, would be desirable, as long as more qualified care conditions would be assured, extending beyond cardiologists, involving trained clinicians and mainly the finally recognized specialists in emergency medicine. Although the emergency medicine specialty already exists in several parts of the world, such as the United States, where the first residency program in the specialty was inaugurated at the Cincinnati University in 1970, it was recognized in Brazil only in 2016.^2^ More recently, the Mixed Committee of Medical Specialties (CME), comprising the Federal Council of Medicine (CFM), the Brazilian Medical Association (AMB) and the National Committee of Medical Residency (CNRM), has put their seal of approval on the education program for emergency medicine, which resulted from the commendable initiative of the Brazilian Association of Emergency Medicine (ABRAMEDE).

It is worth noting that, in the study referred to, although specialized physicians predominated in the care provided to cardiovascular emergencies, both at level II, III and IV hospitals and at SAMU (68.6%), most of them had specialized in areas not related to specific care to cardiovascular diseases, such as pediatrics, general surgery, gynecology and obstetrics, and internal medicine, only 2.9% being cardiologists, while the others had not even attended a medical residency program (31.4%).

Our guidelines for the formation of cardiologists recommend a minimum 288-hour training in cardiovascular emergency.^3^ Other forms of training less directed to that objective, or even the lack of any training, leave a lot to be desired regarding the quality of the care provided to patients with cardiovascular diseases.

In addition, the study referred to evidenced the dissatisfaction with the structure of care provided at cardiovascular emergency units as an important reason for the physicians’ dissatisfaction. However, it is worth highlighting the importance of ‘technical support’ as one of the items related to physicians’ satisfaction, reinforcing the significance of recognizing the area as a relevant element for professional action.

Another element that decisively influences the professional’s satisfaction relates to professional and financial appreciation. Although the topic was not directly assessed by use of the CARDIOSATIS scale,^4^ it is something to be considered in future studies, even for the desired retention of professionals.

This set of measures should be implemented. The practice of medicine amidst such discontentment is inconceivable, mainly at a time with increasing evidence of the significant loss of quality and amount of life among physicians.^5^
